# Optical Properties of Mg, Fe, Co-Doped Near-Stoichiometric LiTaO_3_ Single Crystals

**DOI:** 10.3390/ma5020227

**Published:** 2012-01-30

**Authors:** Wei Tse Hsu, Zhi Bin Chen, Chien Cheng Wu, Ravi Kant Choubey, Chung Wen Lan

**Affiliations:** 1 Department of Chemical Engineering, National Taiwan University, Taipei 10617, Taiwan; E-Mails: hsuweitse@itri.org.tw (W.T.H.); binchenchina@gmail.com (Z.B.C.); b93203021@ntu.edu.tw (C.C.W); ravikantchoubey@gmail.com (R.K.C.); 2 Department of Applied Physics, Birla Institute of Technology, Mesra, Ranchi 835-215, India

**Keywords:** LiTaO_3_ crystals, X-ray diffraction, single crystal growth, holographic properties

## Abstract

Mg, Fe co-doped near-stoichiometric lithium tantalite (SLT) single crystals were grown by employing the zone-leveling Czochralski (ZLCz) technique. The optical properties, holographic parameters, as well as the composition of the grown crystals were measured. It was found that the Li/Ta ratio decreased with the doping of Mg and Fe ions. A red shift was observed in absorption spectrum for the Mg, Fe co-doped crystals compared to the undoped and Mg-doped ones. The effect of the iron ions (Fe^2+^ and Fe^3+^) was further discussed based on the specified absorption bands. Moreover, the occupation mechanism for the defects was discussed by using the IR absorption spectrum, which was attributed to the Fe_Ta_^3−^ defects in the highly Fe-doped crystal. In addition, the holographic parameters were also found to be improved with a higher Fe/Ta ratio in the crystals.

## 1. Introduction

Lithium tantalate (LiTaO_3_, LT) single crystal is an important ferroelectric material for various applications such as second harmonic generation (SHG), surface acoustic wave (SAW) devices, optical modulators, and holographic storage devices [1,2]. Fe-doped congruent lithium tantalate (Fe:CLT) crystal is also a useful material for holographic applications. In the Fe:CLT crystal, during the photo-induced charge transport process, the electrons are redistributed and a space charge field is built up. This occurs because Fe^2+^ and Fe^3+^ ions act as donors and traps, respectively, in the Fe:CLT crystals [3]. The buildup space charge field alters the refractive index of the crystal, so that the Fe:CLT crystal also shows promising photorefractive properties. However, there are still two disadvantages, namely the low damage threshold and the low response speed. Both drawbacks limit the applications of the Fe:CLT crystals in the holographic data storage. Fang *et al*. [4,5] found that the doping of Mg and Zn ions in Fe:CLT might increase the photo-damage resistance and increase response speed. Similar to the lithium niobate (LN) crystal [6], it is believed that the photorefractive properties of LT crystals could be affected by the Li/Ta ratio, which is smaller than unity being about 48.75/51.25 for CLT. The non-stoichiometry in LN introduces anti-site defects that are detrimental to some of the photorefractive properties [7]. In comparison with CLT, the near-stoichiometric lithium tantalate (SLT) crystals also exhibit higher response speed and sensitivity [8]. Recently, we have reported a promising improvement for the Mg, Fe co-doped SLT crystals [9], but the detailed optical properties and the defects were not discussed.

In this report, we further discuss the zone-melting Czochralski (ZLCz) growth [9,10,11,12,13] of Mg, Fe co-doped SLT crystals and their optical properties. With the detailed composition measurements, the defect model would be then discussed based on the absorption spectrum of the grown crystal.

## 2. Experimental Section

Powders of Li_2_CO_3_ (99.995% purity, Honjo chemical Co., Osaka, Japan) and Ta_2_O_5_ (99.995% purity, Taki Chemical Co., Osaka, Japan) were used as raw materials. These raw materials were taken in appropriate proportions and ball milled for 24 hours to prepare a homogeneous mixture. To grow the SLT crystal, a continuous feeding process, the so-called ZLCz method [9,10,11,12,13] was used. The details of the crystal growth and wafer preparation can be found elsewhere [9]. After crystal growth, the wafers were lapped and polished to 1 mm in thickness by a precision lapping and polishing machine (Model PM5, Logitech Co., Palo Alto, CA, USA) for optical measurements. The polished wafers were also annealed at 1,000 °C for 24 h in air atmosphere for oxidization. The heating and cooling rate were controlled at 35~40 °C/h to prevent twinning or cracking of the wafers.

To determine the Li/Ta ratio of the as grown crystals, an ICP-AES (Model ICAP 9000, Thermo Jarrell-Ash Co., Franklin, MA, USA) was used. In this technique the powder samples were fluxed, melted and diluted by DI water [14]. The transmittance and absorbance of the polished wafers were measured by a UV-Visible-NIR Spectrometer (Model V-570, JASCO Co., Tokyo, Japan) and the O-H vibration absorption peak was measured by a Fourier transformation infrared spectrophotometer (Model FT-IR Spectrum One, PerkinElmer Inc., San Jose, CA, USA). The single-crystal XRD measurements were accomplished by using a Siemens D500 diffractometer equipped with a Cu target. The target voltage and current kept at 40 kV and 30 mA, respectively. The lattice parameters were calculated from the X-Ray data.

The holographic data storage properties of these crystals were measured by the two-wave coupling technique. The detail information about the experimental set up can be found in our recent paper [9]. In this experiment, the performance (such as the diffraction efficiency, sensitivity, the dynamic range, *etc*.) of the Mg, Fe co-doped near SLT crystals was measured for holographic storage at room temperature.

## 3. Results and Discussion

### 3.1. As Grown Crystals

**Figure 1:**
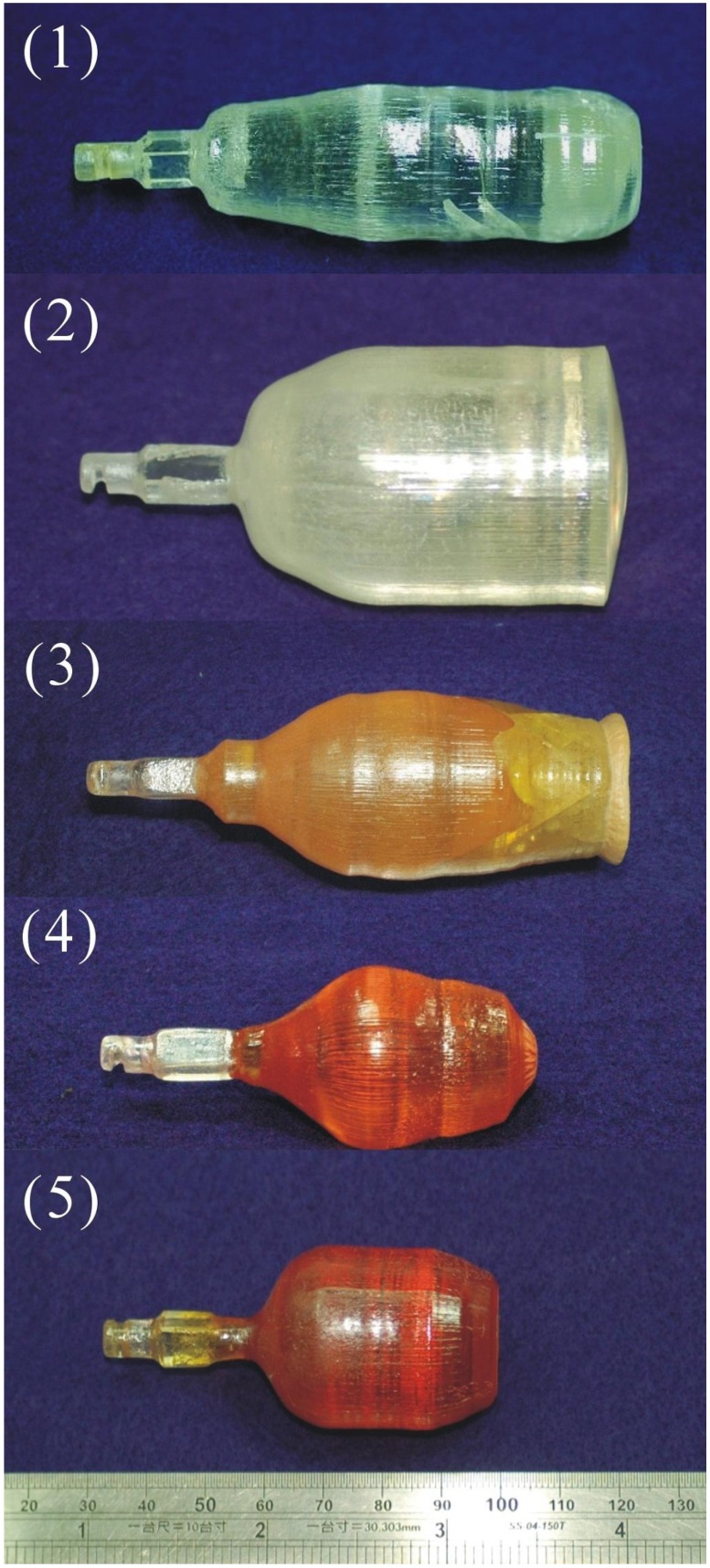
Photograph of the as grown stoichiometric lithium tantalite (SLT) crystals.

The as grown crystals 1 to 5 are shown in Figure 1 and Table 1. It was observed that Crystals 1 and 2 were transparent and bubble free. However, a few small cracks in Crystal 1 and twining on the surface near tail part of Crystal 2 were also observed.

**Table 1:** Growth conditions of SLT crystals.

Crystal no.	1	2	3	4	5
Growth rate (mm/h)	0.6	0.4	0.4	0.2	0.2
Rotating rate (rpm)	6	12	9	9	9
Crystal size (mm^2^)	Φ25 × 75	Φ40 × 60	Φ40 × 70	Φ30 × 50	Φ30 × 50
Li/Ta in solution (-)	1.5	1.5	1.5	1.5	1.5
Mg/Ta in solution (mol%)	0	1.2	1.2	1.2	1.2
Fe/Ta in solution (ppm wt)	0	0	200	1000	2000
Li/Ta in feed (-)	1.041	1.041	1.041	1.041	1.041

After growth, Crystal 1 was cooled from 1,550 °C to 300 °C at a rate of ~35 °C/h and then from 300 °C to room temperature. Because of the fast cooling rate, the induced thermal stress caused the crack in Crystal 1. In Crystal 2, the crystal was pulled out suddenly, so that the growth interface shape could be revealed. A convex surface was observed at the end of crystal, and this was quite typical for the growth from a Li-rich solution. The twining in Crystal 2 was visible in the polished wafers. It was presumed that the absence of the tailing process might enhance the thermal stress in the crystal which caused the twining. The diameters of both the crystals were found to be under good control by using our automatic diameter control (ADC) system. The variation of crystal diameter was observed to be within 1 mm. Crystals 3 to 5 were doped with Mg and Fe, the color of those crystals became darker with the increasing iron amount (as shown in Figure 1). Crystal 3 was found to have constitutional supercooling in its tail and this led to the crack of the crystal. To avoid this, the following crystals (Crystals 4, 5) were grown by modifying two growth parameters. One was to increase the temperature gradient at the solid-liquid interface by reducing the crystal diameter. The smaller crystal diameter enhanced the heat dissipation from the crystal surface leading to a greater temperature gradient. The other one was to decrease the crystal growth rate, which reduced the dopant accumulation in front of the interface. After the new growth parameters were used, crack-free crystals were grown (Crystals 4, 5 in Figure 1). The Proportional–integral–derivative (PID) parameters for ADC were also slightly adjusted in the growth of Crystal 5 to have a good diameter control.

### 3.2. ICP-AES Measurements

The composition of Li/Ta in the as grown crystals was measured by the ICP-AES technique, and the results are shown in the Table 2.

**Table 2:** Composition of the grown crystals measured by ICP-AES technique.

Wafer no.	1	2	3	4	5
Li/Ta (-)	0.995	0.976	0.978	0.981	0.980
Mg/Ta (mol%)	0	1.225	0.926	0.774	0.704
Fe/Ta (ppm wt)	0	0	120	352	650

In Crystal 1, without the doping of Mg and Fe, the Li/Ta ratio was close to unity. However, for the rest of the crystals (Crystals 2 to 5), this ratio was less than unity. It was assumed that the dopants, such as Mg and Fe, replaced the Li sites and thus reduced the Li/Ta ratio. The Li/Ta ratio of Crystals 2 to 5 was about 0.98, which was close to the theoretical limit estimated by the defect model [12]. Table 2 also shows a decrease in the Mg/Ta ratio for Crystals 2 to 5. It was because the MgO powder was added only in the solution zone starting from Crystal 2. As the crystal grew, Mg/Ta ratio was diluted by the feed in the bottom, even though the segregation coefficient of Mg in LT is close to one. To further explain the Mg/Ta ratio, a simple mass balance relation was derived [13]. Moreover, in Crystals 3 to 5, the Fe/Ta ratios, observed in the prepared crystals, were much lower than that in the solution zone. This was due to the evaporation of Fe ion from the solution zone because of its high vapor pressure. Thus with the help of the ICP-AES technique, it was possible to quantify the crystal composition. The correlations between the composition and optical properties in SLT crystals can be investigated.

### 3.3. UV-Visible-NIR Spectrum

**Figure 2:**
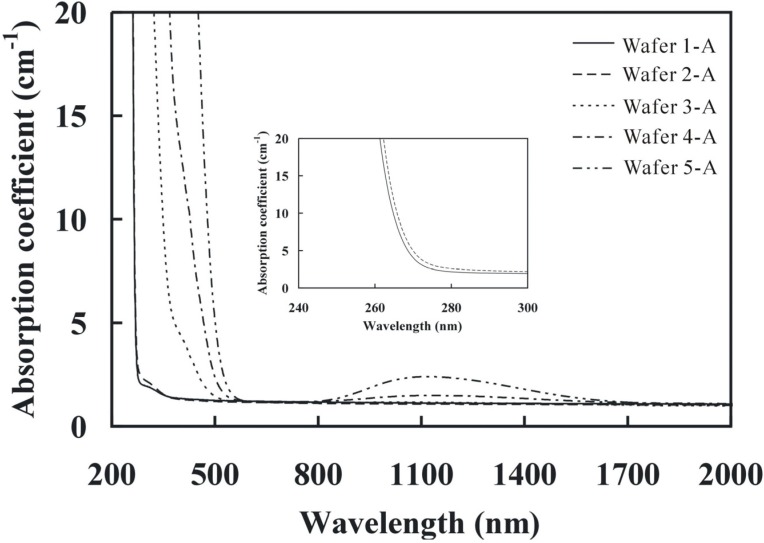
Absorption spectra of the as grown crystals (A).

Figure 2 shows the UV-Visible-NIR absorption spectra of the crystals. The absorption edges of the crystals (Crystals 1 to 5) were measured at the absorption coefficient of 20 cm^−1^, which were 264, 265, 326, 370 and 455 nm, respectively. The absorption edge of Wafer 1-A and 2-A were very close, but for Wafer1-A, it was lower. Since the absorption edge is affected by the Li/Ta ratio [15], therefore, this reduction in the absorption edge was attributed to the high Li/Ta ratio in Wafer 1-A (see Table 2). Also, the wider absorption edge means the narrower energy band gap of the crystal.

In the Mg:Fe:SLT crystals (Wafers 3-A to 5-A), the Li/Ta ratio was about the same but the spectrum still showed some red shift. This shift might be due to the stronger polarizibility effect of the Fe ions, which is greater than that of the Li ions. It is well known that large polarizibility reduces the band gap of the crystal. The absorption edge of Wafers 3-A to 5-A showed a continuous red shift relative to Wafers 1-A and wafer 2-A. Moreover, the Wafers 3-A to 5-A also showed a broader absorption peak in the wavelength of 750 to 1,750 nm and the strongest peak was located at 1,125 nm. The broader absorption peak was believed to be attributed to the d-d crystal field transition of Fe^2+^ [16] and the absorption intensity was proportional to the doped Fe concentration in the crystal.

**Figure 3:**
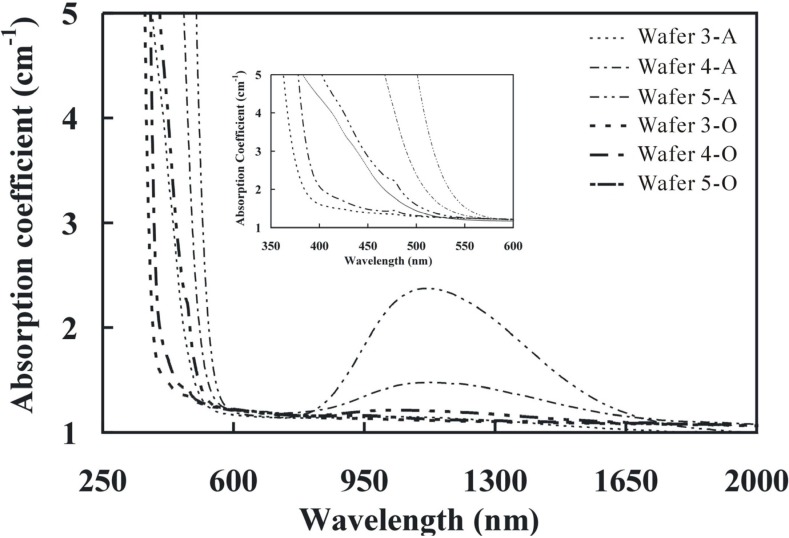
Absorption spectra of the as grown (A) and oxidized (O) crystals.

Figure 3 shows the absorption spectra of the as grown (A) and the oxidized (O) wafers. The absorption edge of the oxidized wafers (Wafers 3-O to 5-O) was 324 nm, 356 nm, and 370 nm, respectively. The oxidation treatment led to a blue shift in the absorption edge as compared with the as grown wafers. As shown in the inset of Figure 3, a narrow absorption band of 476 nm was only observed in the oxidized wafer which was attributed to the d-d transition of Fe^3+^.

It was also assumed that the broad absorption band of 425 nm was due to the inter-valence transfer (Fe^2+^ ➔ Ta^5+^). This broad peak was observed in the Wafer 3-A and Wafer 5-O as shown in the inset of Figure 3. The excess iron amount in the Wafers 4-A and 5-A showed a strong absorption peak, while the low iron amount in Wafer 3A showed a broad absorption band. The inter-valence transfer result showed that there was no complete oxidation of Fe^2+^ to Fe^3+^ in Wafer 5-O. The residual Fe^2+^ ions still produced the broad absorption band in the spectrum. The absorption peak of 425 nm was distinct in our study by controlling the oxidation process.

Figure 3 also showed the broad absorption peaks (of 1,125 nm) in the wafers from the as grown crystals which were much stronger than that in the oxidized wafers because this broad absorption band was also affected by Fe^2+^ ions. The absorption peak (Figure 3) in Wafer 5-O showed slightly stronger than that in Wafers 3-O and 4-O, which was again attributed to the existence of the residual Fe^2+^ ions.

### 3.4. OH^−^ Absorption Spectrum

**Figure 4:**
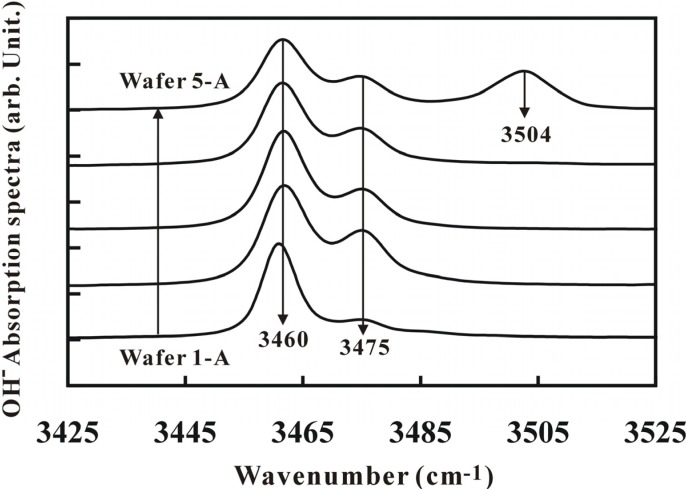
Infrared absorption spectra of the as grown crystals.

Figure 4 showed the OH^−^ absorption spectrum of LT crystals. In the LT crystals, the ionic bonding of Ta-O was much stronger than that of Li-O. Due to the stronger ionic bonding of Ta-O, some Ta ions occupied Li sites forming the anti-site defect of Ta_Li_^4+^ and Li vacancies (V_Li_^−^).

It is well known that H^+^ ions might replace the Li ions or attach with O-O bonding positioning themselves at the interstitials [17,18]. Therefore, H^+^ ions were easily attracted by the V_Li_^−^ defects because of the different electrical charges and the formation of (V_Li_)^−^OH^−^ complexes. These complexes showed the absorption peak at 3,475 cm^−1^ in the IR spectrum [17]. For the SLT crystal, the anti-site defect was less; therefore, we assume that the majority of H^+^ ions were attracted to the Li sites generating the vibrational mode of Li-OH^−^. The absorption peak for Li-OH^−^ vibration was located at 3,460 cm^−1^. The absorption peak height ratio for Li-OH^−^ and V_Li_^−^OH^−^ (*i.e*., Li-OH^−^/V_Li_^−^OH^−^) from IR spectrum for Wafers 1-A to 5-A at the wave numbers of 3,460 cm^−1^ and 3,475 cm^−1^, respectively, also showed the evidence that they could be used to judge the Li/Ta ratio. From Figure 4, the Li-OH^−^/V_Li_^−^OH^−^ ratio (which means the Li/Ta ratio) for Wafer 1-A was larger than the others. This result was consistent with the Li/Ta ratio for Wafer 1-A from the ICP-AES measurement. Moreover, the absorption spectrum for Wafer 1-A also showed a red shift in the Li-OH^−^ mode from the OH^−^ spectrum analysis relative to other crystals and the red shift in the OH^−^ absorption peak for Wafer 1-A was also an evidence for its higher Li/Ta ratio. The peak ratio and the peak shift (red shift) in the OH^−^ absorption spectrum for all wafers showed the same trend as in the ICP-AES measurements. However, the absorption peak of V_Li_^−^OH^−^ showed no effects on the Li/Ta ratio in the SLT crystal [19] and all peaks are located at wave number of 3,475 cm^−1^. It meant that the peak corresponding to V_Li_^−^OH^−^ was less sensitive in the Li/Ta ratio as this ratio was closer to the stoichiometry. A new absorption peak at the wave number of 3,504 cm^−1^ in Wafer 5-A was observed in addition. This peak appeared only in Wafer 5-A because of the threshold doping of Fe into it to form Fe_Ta_^3−^ ions. Liu *et al*. [20] have observed that the Fe ions were more active than the Mg ions to replace Nb ions in the Mg:Fe-codoped LN crystal and Mg_Li_^+^-OH^−^-Fe_Nb_^3−^ bonding showed an absorption peak at 3,504 cm^−1^. Thus, it is clear in our study that this additional peak in Wafer 5-A could be due to the Ta site being replaced by Fe ions. This peak (in Wafer 5-A) corresponds to wave number 3,504 cm^−1^ might be due to the Mg_Li_^+^-OH^−^-Fe_Ta_^3−^ bonding. However, no peaks were observed due to Mg_Ta_^3−^ ion in the OH^−^ spectra because the Mg concentration did not reach the doping threshold of forming Mg_Ta_^3−^ ions and thus the Mg_Li_^+^-OH^−^-Mg_Ta_^3−^ bonding did not take place. As a result, no absorption peak corresponding to Mg_Ta_^3−^ ions was observed.

### 3.5. X-ray Diffraction (XRD) Measurements

**Figure 5:**
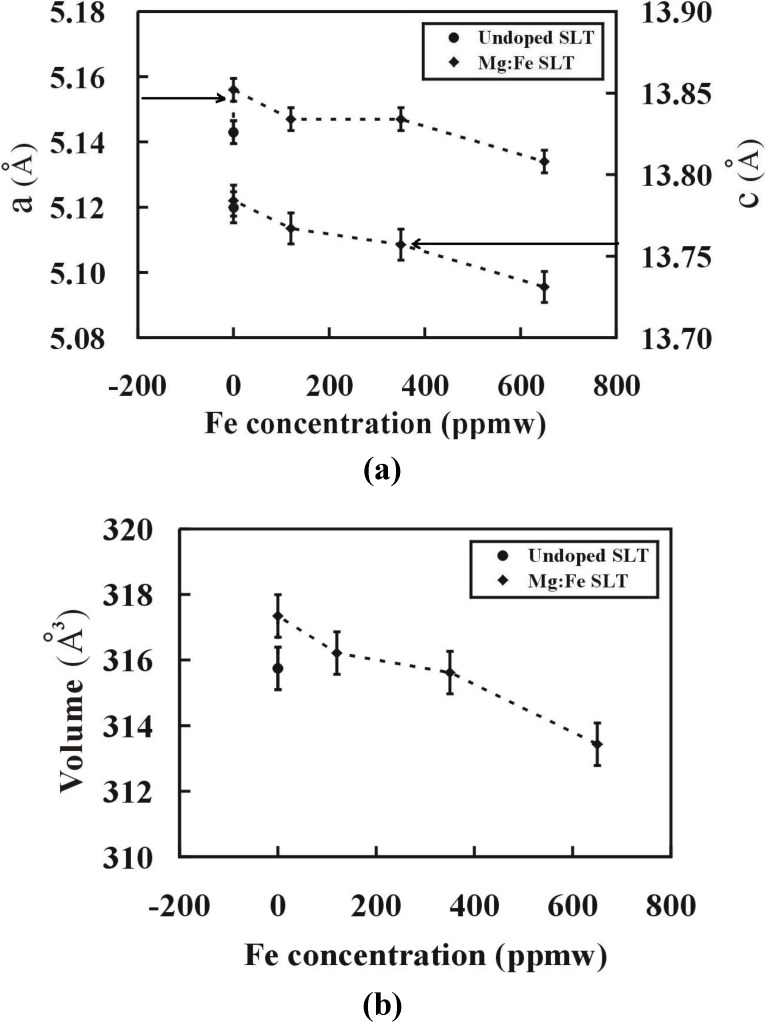
Variation of (**a**) lattice constants and (**b**) volume of the unit cell with the increasing Fe concentration in the crystals.

The X-ray diffraction analysis results indicate that Mg doped and Mg, Fe codoped LT crystals keep the same structural characteristics as pure LT, but the lattice constants (a and c) and the volume of lattice cell change (see Figure 5). The differences in the lattice constants between doped and pure LT indicate the degree of lattice distortion in the crystal.

From Figure 5, it is observed that both (lattice constants and volume) increased by only Mg doping while after adding Fe ion as co-dopant both parameters decreased with the increasing Fe concentration in the crystal. The change in these parameters depended on the Li/Ta ratio in the crystal. It can be observed that it was inversely proportional to the Li/Ta ratio in the crystals. Only the Mg doping reduced the Li/Ta ratio, so that the subsequent increase in the lattice constants and volume could be observed. By the co-doping of Fe ions in the crystals, the Li/Ta ratio increased as compared to Mg doped SLT crystals, and both the parameters decreased. In other words, the unit cell was expanded by the Mg doping and the co-doping with Fe ions contracted the unit cell.

### 3.6. Holographic Properties of Mg, Fe Co-Doped Near SLT Crystals

The holographic properties of crystals were also measured and the results are listed in Table 3.

**Table 3:** Variation in photorefractive properties of the Mg, Fe co-doped near-SLT wafers under different Fe concentrations.

At Intensity 1 W/cm^2^		
Holographic Parameters	Wafer-3 (200 ppm Wt)	Wafer-4 (1000 ppm Wt)
η_max_ (%)	14.3	33.4
Δn (10^−5^)	3.17	5.03
τ_r_ (S)	31.3	34.5
τ_e_ (S)	95.1	267.9
M (#)	0.9	3.4
S (10^−2^ cm/J)	2.4	3.2

The diffraction efficiency (η), which represents the “strength” of the hologram, is an important parameter for crystals used in the holographic storage. The higher diffraction efficiency indicates the better energy transferability. Larger diffraction efficiency is also better for holographic storage applications. The diffraction efficiency can be written as

(1) $$\eta =\frac{I_{d}}{I_{r}+I_{d}}\times 100\%$$

where *I_d_* is the diffracted light intensity and *I_r_* is the light intensity of transmitted reference beam. The photorefractive index change Δ*n* can be obtained using the Kogelnik’s formula [21].

(2) Δn=λcosθsin−1(η12)πL

where *L* is the wafer thickness, θ is the incident angle inside the crystal and λ is the incident wavelength. The calculated values of Δ*n* are given in the Table 3 and it is clear that they are related to diffraction efficiency. The refractive-index change Δ*n* is related to the space charge field *E_SC_* as

(3) $$\Delta n=-\frac{1}{2}{n_{0}}^{3}r_{eff}E_{SC}=-\frac{1}{2}{n_{0}}^{3}r_{eff}\frac{j_{ph}}{\sigma }\approx \frac{k\alpha 1}{\sigma_{d}+\sigma_{ph}}$$

where r_eff_ is the effective electro-optic coefficient, n_0_ is the effective-index, j_ph_ is the photovoltaic current, *k* is the glass constant, *I* is the light intensity, and σ_d_ and σ_ph_ are the dark and photoconductivity of the crystal, respectively.

Here, the diffraction efficiency of Wafers 3 and 4 is discussed at an intensity of 1 W/cm^2^. From the result, it is clear that the diffraction efficiency of Wafer 4 was much larger than that of Wafer 3. In congruent LT crystals, the anti-site Ta_Li_ defects form defect energy grade in the forbidden band of this crystal and play the role of photorefractive centers during holographic recording. Since much less intrinsic defects exist in the pure near-SLT crystal, this result in a small number of photorefractive centers and increase the photoconductivity [22]. Consequently, the pure SLT crystal has the poor holographic storage properties as a whole [23]. However, in the case of Mg, Fe-codoped SLT crystals, the doping of Mg ion enhanced the photoconductivity in the crystal. The Mg/Ta ratio of Wafer 3 was larger than that of Wafer 4. Still, wafer 4 showed higher diffraction efficiency than Wafer 3 suggesting that Fe ions play a more important role as compared to Mg ion in the influence of diffraction efficiency. With the increasing Fe doping, the photoconductivity significantly decreased; therefore, the diffraction efficiency was larger in Wafer 4 as compare to Wafer 3. Since, Δ*n* is related to diffraction efficiency, so it might follow the same trend. Consequently, the change in refractive index in Wafer 4 is larger as compared to that in Wafer 3.

The recording time constant (τ_r_) and the erasing time constant (τ_e_) of Mg, Fe co-doped near-SLT crystals were measured using the two-beam coupling technique. The recording time constant is defined as the time when the diffraction efficiency reaches η_max_ (1 − e^−1^) from zero, while the erasing time constant is defined as the time when the diffraction efficiency decreases to η_max_ (e^−1^) from η_max_ The symbol η_max_ denotes the maximum (saturation) diffraction efficiency. From Table 3 it can be observed that recording as well as erasing time constants of Wafer 4 was larger as compared to Wafer 3. Since τ_r_ and τ_e_ both are directly proportional to the diffraction efficiency, the recording time constant and the erasing time constant were smaller in Wafer 3. The reason for the smaller τ_r_ and τ_e_ in Wafer 3 is same as explained above for the diffraction efficiency.

Two of the most important parameters of photorefractive materials for the holographic storage systems are the sensitivity (S) and dynamic range (M/#). The sensitivity of the crystal describes how much optical energy is needed to produce a given change in the refractive index. High sensitivity is always favorable for recording holograms, since if the crystal has a higher sensitivity; it means that it has a shorter writing time. In the single-hologram recording and erasure experiments, the sensitivity can be calculated as follows

(4) S=(∂η∂t)t=0IL

The dynamic range (M/#) is another important parameter for photorefractive materials. A larger dynamic range means that the crystal has a higher storage density and a better signal-to-noise ratio. The dynamic range coefficient M/# can be defined by the following formula

(5) $$M={\left(\frac{\partial \sqrt{\eta }}{\partial t}\right)}_{t=0}\times \tau_{\theta }$$

Using relation (4) and (5) above, we can now calculate the sensitivity and dynamic range of the Mg, Fe co-doped near SLT crystals. Both the sensitivity and dynamic range of the crystal are proportional to $\frac{\partial \sqrt{\eta }}{\partial t}$. The sensitivity and dynamic range of Wafer 4 was larger than Wafer 3, which was attributed to the higher Fe concentration in Wafer 4. Recently, Zhang *et al*. [23] have reported the value of sensitivity and dynamic range of 0.00928 cm/J and 1.68, respectively, in Mn doped SLT crystals. We have achieved a larger value in our Mg, Fe co-doped near SLT crystals.

## 4. Conclusions

The optical and holographic storage properties of Mg, Fe co-doped near-stoichiometric lithium tantalate single crystals have been investigated. The results were discussed in terms of the Li/Ta ratio, which was related with the absorption edge as well as the energy band gap of the crystals. A broad absorption band (of 425 nm) was observed only in a certain amount of Fe^2+^ ions. The OH^−^ spectrum analysis showed that the Li-OH^−^ vibration dominated over the V_Li_^−^OH^−^ one because of the less vacancy sites in the crystal. A new peak at 3,504 cm^−1^ in the OH^−^ spectra indicated the presence of the Mg_Li_^+^-OH^−^-Fe_Ta_^3−^ bonding. It was observed that the energy band gap of the SLT crystals became narrower due to the presence of Fe ions in the crystal which led to a red shift in the absorption spectrum. The XRD analysis confirmed that the basic crystal structure was the same after doping and the lattice parameters were found to decrease due to the Fe doping. The holographic properties of the crystals were greatly improved with the co-doped SLT crystals as compared to the reported one.
